# Improving the Effect of Transcranial Alternating Current Stimulation (tACS): A Systematic Review

**DOI:** 10.3389/fnhum.2021.652393

**Published:** 2021-06-07

**Authors:** Linyan Wu, Tian Liu, Jue Wang

**Affiliations:** ^1^The Key Laboratory of Biomedical Information Engineering of Ministry of Education, Institute of Health and Rehabilitation Science, School of Life Science and Technology, Xi’an Jiaotong University, Xi’an, China; ^2^National Engineering Research Center of Health Care and Medical Devices, Guangzhou, China; ^3^The Key Laboratory of Neuro-informatics & Rehabilitation Engineering of Ministry of Civil Affairs, Xi’an, China

**Keywords:** transcranial alternating current stimulation, high definition electrical stimulation, phase-shifted tACS, amplitude modulated tACS, temporally interfering method, intersectional short pulse method

## Abstract

With the development of electrical stimulation technology, traditional transcranial alternating current stimulation (tACS) technology has been found to have the drawback of not targeting a specific area accurately. Studies have shown that optimizing the number and position of electrodes during electrical stimulation has a very good effect on enhancing brain stimulation accuracy. At present, an increasing number of laboratories have begun to optimize tACS. However, there has been no study summarizing the optimization methods of tACS. Determining whether different optimization methods are effective and the optimization approach could provide information that could guide future tACS research. We describe the results of recent research on tACS optimization and integrate the optimization approaches of tACS in recent research. Optimization approaches can be classified into two groups: high-definition electrical stimulation and interference modulation electrical stimulation. The optimization methods can be divided into five categories: high-definition tACS, phase-shifted tACS, amplitude-modulated tACS, the temporally interfering (TI) method, and the intersectional short pulse (ISP) method. Finally, we summarize the latest research on hardware useful for tACS improvement and outline future directions.

## Introduction

Transcranial electrical stimulation is an established option for inducing neuroplasticity and modulating cortical function. At present, transcranial alternating current stimulation (tACS) as a method to regulate intrinsic nerve concussion by applying a weak alternating electric field has attracted increasing attention (Dayan et al., 2013). The two most important factors affecting the clinical application of tACS are the limited intensity of stimulation and the difficulty of accurately focusing the stimulating electric field. This is also a common problem of transcranial electrical stimulation. A large part of the current injected during electrical stimulation will be shunted by the scalp, bypassing the brain completely and limiting the electric field intensity of the target area (Dmochowski et al., 2011). In view of this disadvantage, optimizing the number and position of electrodes is an effective means. Different studies have proven that changing electrode properties or changing current properties can improve the degree of focus during stimulation (Huang and Parra, 2018; Saturnino et al., 2019). At present, the common optimization methods for tACS can be divided into high-definition electrical stimulation methods and interference modulation methods. The high-definition electrical stimulation method replaces large electrodes placed in anatomical order with several small electrodes to enhance the focus effect (Kuo et al., 2013; Reinhart, 2017). The interference modulation method generates a specific waveform at a specific position in the brain through the mutual interference of two or more electrode signals to meet the requirements of non-invasive deep brain stimulation (Grossman et al., 2017; Kasten et al., 2018; Alekseichuk et al., 2019b). Therefore, the main purpose of this review is to discuss the differences of the above tACS optimization methods to compare the effects of different improvement methods in neuromodulation.

Herein, we review all studies that have applied optimized tACS methods to provide a comprehensive perspective of past and current research. Then, we assess the possible differences between these specific electrical stimulation schemes, which can help to determine the potential therapeutic effects of different electrical stimulation methods in future studies. In addition, because high-definition electrical stimulation has a specific demand for electrode materials and other characteristics and because interference modulation sometimes requires a high-frequency waveform or other specific waveforms, this review also discusses some hardware that can be used in these improved stimulators.

## Optimization of Tacs

**Figure 1:**
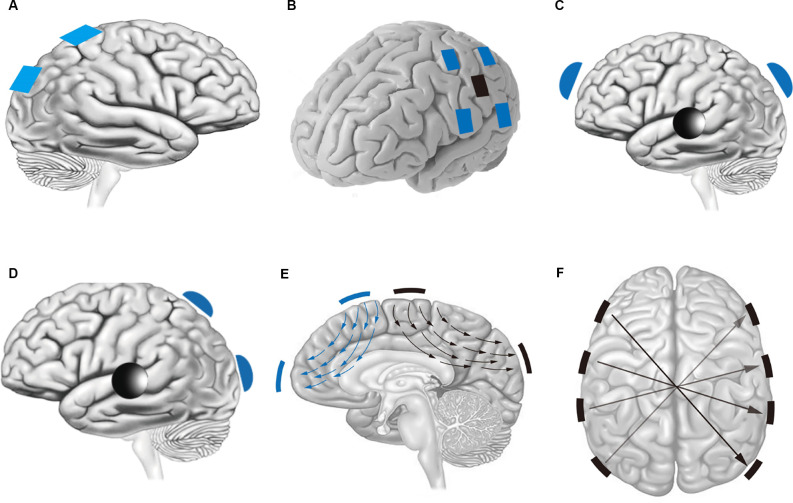
**(A)** In traditional tACS, the position of the electrode is determined by the location of the stimulation. **(B)** HD-tACS; blue regions are the anodes, and the black region is the cathode. **(C)** Phase-shifted tACS; blue regions are the anodes, and the black region is the common cathode; the stimuli exerted by the two blue electrodes are only different in phase. **(D)** AM-tACS; blue regions are the anodes and the black region is the common cathode; the stimuli exerted by the two blue electrodes are, respectively, the envelope wave and the modulated wave. **(E)** Temporally interfering (TI) stimulation; the blue and black regions are two pairs of electrodes with different high-frequency electrical stimulations. **(F)** Intersectional short pulse (ISP) stimulation; a pair of electrodes at both ends of each arrow, and the stimulation time of each pair of electrodes is different.

In recent years, because cathode/anode/double electrode stimulation cannot effectively control the stimulation focus and intensity, people have begun to change the electrode position and current properties to optimize focus and achieve higher-intensity stimulation (Douglas et al., 2015). Because of the dispersion of electric currents in biological tissues, it is challenging to achieve high spatial precision. The stimulation region of tACS (Figure 1A) is also constrained to the area under the stimulation electrode, and the stimulation to the brain is diffuse; that is, it is impossible to achieve precise stimulation for a certain deep brain region. After studying the stimulation electrodes and stimulation current of tACS, the improvements can be divided into the following two types. One improvement is to simply split the large electrode into several small electrodes during stimulation to enhance the focusing effect by the joint action of several small electrodes, which is called high-definition electrical stimulation (HD-tACS; Figure 1B). Another improvement is achieved by changing the stimulated current mode to optimize the focus and enhance the intensity of electrical stimulation; we call this version interference modulation electrical stimulation. Interference-modulated electrical stimulation can be divided into five types: phase-shifted tACS (Figure 1C), amplitude-modulated tACS (AM-tACS; Figure 1D), the temporally interfering (TI) method (Figure 1E), and the intersectional short pulse (ISP) method (Figure 1F).

### High-Definition Electrical Stimulation

**Figure 2:**
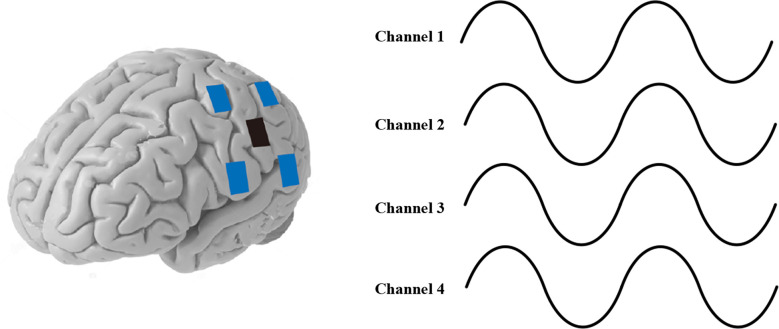
Schematic diagram of HD-tACS.

The principle of HD-tACS, like that of tACS, is stimulation of a single brain region. Its principle is to enhance the amplitude of brain electrical signals in a specific frequency band by coupling the frequency of the stimulating current with the frequency of neuronal activity in the brain to achieve an intervention effect. As shown in Figure 2, HD-tACS applies a constant amplitude of weak current to the brain mediated by electrodes attached to the scalp. Then, it can change the electrical activity of the brain and achieve neural regulation of the brain. Blue electrodes are connected with the positive current source, and the black electrode is connected with the negative current source, or vice versa. The typical stimulation parameters were the same as the tACS stimulation parameters, which were a 1–2 mA stimulation current, alpha band or theta band stimulation frequency, and continuous stimulation for 20 min. Based on the available literature, we summarized the parameters of high-definition electrical stimulation in different groups, as shown in Table 1.

**Table 1:** High-definition electrical stimulation.

Reference	Sample size (*n*)	Stimulation type	Parameter location	Parameter intensity	Duration	Main outcome
Lang et al. (2019)	60 (age: 18–45 years)	HD-tACS	FP1, P2, P3, PO7, P10	6 Hz, Fp1: −0.62 mA, P2: −0.99 mA, P3: −0.14 mA, P7: −0.24 mA, P10: 2 mA (peak to baseline)	10 min	Consistent with our hypothesis, improved AM performance was observed in the TACS group, while TDCS had no effect.
Berger et al. (2018)	24 (12 females, 12 males, age: 18–30 years)	HD-tACS	P3, P4	10 Hz/20 Hz, 1 mA	20 min	After HD-tACS stimulation, the alpha activity in the parietal lobe increased significantly, and the concentration of HbOxy in right hemisphere motor cortex decreased significantly.
Wischnewski et al. (2018)	11 (9 females, 2 males, age: 23.1 ± 3.4)	HD-tACS	C3, T7, F3, Cz, P3	20 Hz, 2 mA	15 min	tACS can induce NMDAR-mediated plasticity in the motor cortex.
Alekseichuk et al. (2017)	25 (13 females, 12 males, age: 23.5 ± 2.9 years)	HD-tACS	AF3, P3, AF4, P4	6 Hz, 1 mA (peak to baseline)	18 min	A decrease in memory performance and an increase in reaction time was caused by frontoparietal intra-hemispheric desynchronization.
Alekseichuk et al. (2019b)	25 (13 female, 12 males, age: 18–28 years)	HD-tACS	P4, T8, C2, CP1, Oz	4 Hz, 3 mA	20 min	Stimulation over the right posterior brain area augmented subsequent long-term recognition memory.
Khatoun et al. (2018)	13 (4 females, 9 males, age: 24 ± 5)	HD-tACS	C3, C1, C5, CP3, FC3/C4, C2, C6, CP4, FC4/Pz, Oz, Pz, PO4, PO3	10 Hz, 5 mA	12 min	High-amplitude focused HD-tACS can entrain physiological tremor.
Reinhart (2017)	97 (45 females, 52 males)	HD-tACS	MFC, lPFC	6 Hz, 1 mA	20 min	Executive functions can be rapidly up- or downregulated by modulating theta phase coupling of distant frontal cortical areas
Zoefel et al. (2019)	19 (8 females, 11 males, age: 21 ± 2 years)	HD-tACS	T7, T8	3.125 Hz, 1.2 mA	19 min	tACS induces speech perception modulation, but only if the stimulation was applied bilaterally using ring electrodes (not for unilateral left hemisphere stimulation with square electrodes).
Helfrich et al. (2014)	14 (8 females, 6 males, age: 27.5 ± 6.7 years)	HD-tACS	C3, C4, O1, O2/P7, PO7, P8, PO8	40 Hz, 1 mA	20 min	In-phase stimulation enhanced synchronization of inter-hemispheric functional connectivity, and anti-phase stimulation impaired functional coupling.
Schwab et al. (2019)	24 (12 female, 12 males, age: 26 ± 4)	HD-tACS	P7, PO7, P8, PO8	9.5–10.5 Hz, 2 mA	13 min	Global pre–post stimulation changes in EEG connectivity were larger after in-phase stimulation than after anti-phase or jittered-phase stimulation.
Popp et al. (2019)	28 (14 females, 14 males, mean age: 24.4 years)	HD-tACS	C3, C4, CP3, CP4, P3, P4/T7, T8, TP7, TP8, P7, P8	1–7.5 Hz, 1 mA	20 min	TACS and sham conditions did not differ regarding their reaction times in response to target stimuli or their event-related spectral perturbation (ERSP) at stimulation frequency.
Tomer et al. (2018)	62	HD-tACS	F3, F4, C3, C4, P3, P4, O1, O2	Determined by a staircase procedure	20 min	Alpha oscillations did not increase after HD-tACS relative to the sham condition.
Nguyen et al. (2018)	32 (16 females, 16 males, mean age: 24 years)	HD-tACS	MFC, right lPFC	6 Hz, 1 mA	20 min	HD-tACS with participants’ eyes open improved learning ability relative to sham stimulation, whereas HD-tACS with participants’ eyes closed had no significant effect on behavior.
Deng et al. (2019)	38 (28 females, 10 males, age: 18–24 years)	HD-tACS	P2, CP2, P4, Pz, PO4	10 Hz/6 Hz, 1.5 mA	20 min	Parietal alpha brain stimulation affects top-down control of auditory spatial attention with causal, frequency, hemispherical and task-specific effects.

According to the location of stimulation, HD-tACS can be divided into unilateral stimulation and bilateral stimulation. High-amplitude focused unilateral tACS can lead to physiological tremor (Khatoun et al., 2018). This is also the effective mechanism of HD-tACS. In the theta band, 6 Hz is a commonly used stimulus frequency. Unilateral HD-tACS of 6 Hz on the medial frontal cortex (MFC) and left prefrontal cortex (LPFC) can influence executive function by regulating the coupling of theta waves in the distal frontal cortex (Reinhart, 2017). Pertinently, unilateral HD-tACS of 6 Hz can also affect visual spatial working memory by reducing the phase connectivity between the MFC and LPFC (Alekseichuk et al., 2017). In addition, unilateral HD-tACS has also been proven to be able to improve learning according to the brain state and improve associative memory (AM) performance (Nguyen et al., 2018; Lang et al., 2019). In addition to the commonly used 6-Hz frequency stimulation, people have also studied stimulation at other frequencies. Unilateral HD-tACS (4 Hz) on the right posterior region of the brain can enhance long-term recognition memory (Alekseichuk et al., 2019c). HD-tACS (10 Hz) of the parietal lobe influences top-down control of auditory spatial attention and can also cause electrophysiological and hemodynamic changes in the motor network of the brain (Berger et al., 2018; Deng et al., 2019). HD-tACS (20 Hz) in the M1 region can induce NMDAR-mediated motor cortex plasticity. Aside from the use of bilateral stimuli and the replacement of standard rectangular electrodes with ring electrodes, which has been shown to improve the focus and efficiency of stimuli, there are few studies on bilateral HD-tACS (Datta et al., 2009; Saturnino et al., 2015; Heise et al., 2016). Zoefel found that HD-tACS modulates language perception, but only in the case of bilateral stimulation with a circular electrode (rather than unilateral left hemispheric stimulation with a square electrode; Zoefel et al., 2019). Similar to 6-Hz unilateral HD-tACS, 40-Hz bilateral HD-tACS can also modulate the interhemispheric gamma frequency band of perceptual correlation and other neural activities (Helfrich et al., 2014; Schwab et al., 2019).

Recently, the shape and number of electrodes of HD-tACS have been gradually optimized; the traditional 4 × 1 electrode placement method and traditional circular electrodes are no longer used. Lafon compared the effects of trapezoidal electrodes, strip electrodes, grid electrodes, and depth electrodes, as well as unilateral tACS stimulation and bilateral tACS stimulation. However, the author did not obtain a significant result in this comparison (Lafon et al., 2017). In other words, although HD-tACS can improve the stimulation effect, the electrode shape used for HD-tACS and unilateral or multiple HD stimulation have no effect on the final stimulation effect. In addition, some researchers have explored the stimulation mode of 2 × 6 electrodes and reversed-phase eight electrodes, but their good effects have not been obtained (Tomer et al., 2018; Popp et al., 2019). In other words, the number of electrodes in HD-tACS has no effect on the final effect of stimulation. From the above research, the number of electrodes, the shape of the electrode, and the stimulation mode have no significant effect on the final stimulation effect; therefore, why the effect of HD-tACS is better than that of ordinary tACS is still a question worth studying.

### Interference Modulation Electrical Stimulation

In addition to simply replacing the large electrode with several small electrodes, people have also improved the tACS from the perspectives of the amplitude, frequency, and phase of the stimulating current. The optimization of these aspects can be called interference modulation. Interference modulation is a way to make the stimulation signals of two or multiple electrodes interfere with each other to modulate the signal into a specific waveform at a specific region. At present, the main methods to achieve accurate stimulation through interference modulation are phase-shifted tACS, AM-tACS, TI stimulation, and ISP stimulation. Based on the available literature, we summarized the parameters of interference modulation electrical stimulation in different groups, as shown in Table 2.

**Table 2:** Interference modulation electrical stimulation.

Reference	Sample	Stimulation type	Parameter location	Parameter stimulation	Duration	Main outcome
Alekseichuk et al. (2019a)	Two non-human primates	Phase-shifted tACS	Middle forehead, left occipital area, left temporal area	0.1 mA (peak-to-baseline), 10 Hz, the phase of the stimulation currents starts from 0° phase up to 360° in 15° steps.	Each stimulation condition lasted for 30 s with a ramp up/down time of 5 s	Describe a previously unreported capability of multi-electrode TACS to generate “traveling wave” electric fields in the brain.
Haslacher et al. (2020)	7 humans (3 females, 4 males, age: 22–28 years)	AM-tACS	Over CPz and below Oz	2 mA, 500 Hz carrier and 10 Hz envelope signal	4.5 min	SASS can be used to establish adaptive (closed-loop) AM-tACS
Grossman et al. (2017)	Mouse: C57BL/6 B and CK-p25	Temporally interfering stimulation	AP: −2 mm, ML: −0.25 mm, AP: −2 mm, ML: 2.75 mm	2 kHz/2.01 kHz, 125 μA,	20 min	By altering the currents delivered to a set of immobile electrodes, we can directly evoke different motor patterns in living mice.
Vöröslakos et al. (2018)	16 females, 3 males Long-Evans rats, 8 male Wistar rats	Intersectional short pulse stimulation	3 mm posterior from Bregma and 2 mm lateral of the midline	Varying frequencies (10, 100, and 1,000 Hz) at varying amplitudes (10, 20, 50, 100, and 200 μA)		When high-intensity current is injected into the brain, the charge density and sensation on the scalp surface are relatively low.
Vöröslakos et al. (2018)	19 healthy humans	Intersectional short pulse stimulation	12 stimulation electrodes (six on each side) were placed around the head	ISP stimulation consisted of a train of 1-Hz sinusoids with increasing and decreasing intensity (0, 1.5, 3, 4.5, 6, 7.5, 6, 4.5, 3, 1.5, or 0 mA per cycle)	720 s	ISP stimulation at sufficient stimulus intensity (4.5–6 mA) can bring about stimulus phase-dependent amplitude modulation of the EEG signals.

### Phase-Shifted tACS

**Figure 3:**
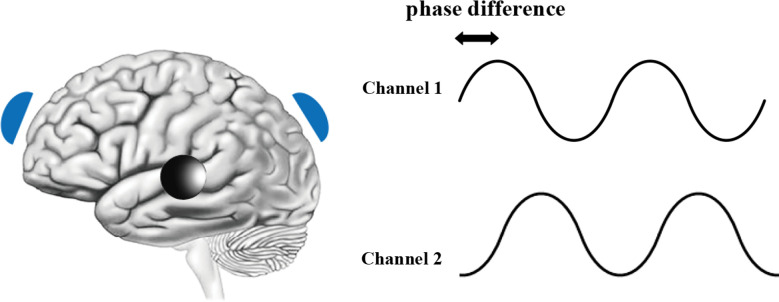
Schematic diagram of phase-shifted tACS.

If we improve the tACS of traditional single brain regions, it is easy to think of a way to increase the number of brain regions stimulated by electrical stimulation. Therefore, when double brain region stimulation occurs, even if the two AC signals have the same amplitude and frequency, the phase of transcranial stimulation is difficult to guarantee. The application of tACS in multiple brain regions depends on the assumption that the phase shift between stimulus currents can be linearly transformed into the phase difference between electric fields generated in various locations of the brain (Alekseichuk et al., 2019b). This hypothesis has been proven to be suitable only for the case of double electrodes (Opitz et al., 2016), and phase-shifted tACS was proposed on this basis. Phase-shifted tACS is essentially the stimulation of two brain regions. It is mainly used to change the connectivity of various brain regions. The principle, like tACS, is to interfere with brain electrical signals in a particular frequency band by applying the frequency of the stimulating electrode. At the same time, due to the difference in the phase of the stimulation current, phase-shifted tACS is influenced by the interval between the activation time of different stimulation regions; that is, different stimulation regions are activated in turn according to the phase of the stimulation, thus affecting the connectivity of each stimulated region of the brain. As shown in Figure 3, two blue stimulation electrodes cover two target brain regions, and a third black electrode acts as a return electrode outside the region of interest (Alekseichuk et al., 2019c). The typical stimulus parameters are the same as those of tACS and HD-tACS; the only disparity is that the phase difference of the two stimulus signals is 0°–360° (usually the difference is between 180° and 90°). Several teams have adopted this concept (Violante et al., 2017; Tseng et al., 2018) or optimized it (Ströber et al., 2014; Alekseichuk et al., 2017). Recently, Alekseichuk studied different phases of phase-shifted tACS and found that multielectrode and polyphase tACS can produce “traveling wave” stimuli. The location of the largest stimulus changes with time, and the magnitude of the electric field is different under different stimulation phases (Alekseichuk et al., 2019b). However, the study of Alekseichuk also has some shortcomings. Kadir pointed out that in some phase changes and delays, nerve stimulation has the same effect as sham stimulation. This requires us to further subdivide the phase differences between stimuli (Kadir et al., 2020).

### Amplitude Modulation tACS

**Figure 4:**
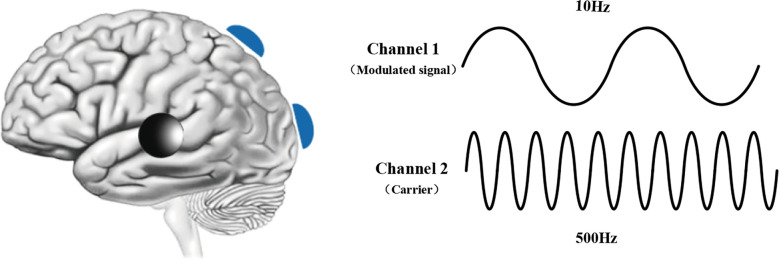
Schematic diagram of AM-tACS.

From phase-shifted tACS, we wonder if the electrodes are placed in the same way, can the electrodes stimulate the same brain region? Combined with the knowledge of FM and AM in the field of communication, Bachinger proposed the concept of power synchronous tACS; that is, the tACS signal fluctuates in amplitude according to the power envelope of the lower frequency signal (1 Hz). Power synchronous tACS significantly increases the connectivity of rs-fMRI in the stimulated resting state network (Bächinger et al., 2017). Based on this study, the concept of amplitude modulation transcranial alternating current stimulation (AM-tACS) was proposed. It is a new method to suppress stimulation artifacts (Kasten et al., 2018). The principle of this stimulation method is exactly the same as that of tACS, but it is convenient for us to deal with the artifacts of the collected signals. As shown in Figure 4. AM-tACS and phase-shifted tACS are the same. They require the placement of three electrodes. The difference lies in the frequency of stimulus signals. In scientific research, it is often the goal to collect the subjects’ EEG data at the same time as electrical stimulation. However, when tACS stimulation and EEG acquisition are carried out at the same time, the artifact of electrical stimulation seriously interferes with the collected EEG, and the actual EEG signal is submerged by stimulation artifacts; that is, it is difficult to simultaneously collect traditional tACS and EEG data simultaneously (Negahbani et al., 2018). The principle of AM-tACS is that a low-frequency envelope is caused by the interference of two sine waves of different frequencies. The typical stimulation parameter of AM-tACS is continuous stimulation for 20 min by a 1- to 2-mA current. AM-tACS is mainly used in the study of brain function rather than clinical treatment. At present, whether it can effectively reduce the influence of artifacts on signal processing is still under discussion.

### TI Stimulation

**Figure 5:**
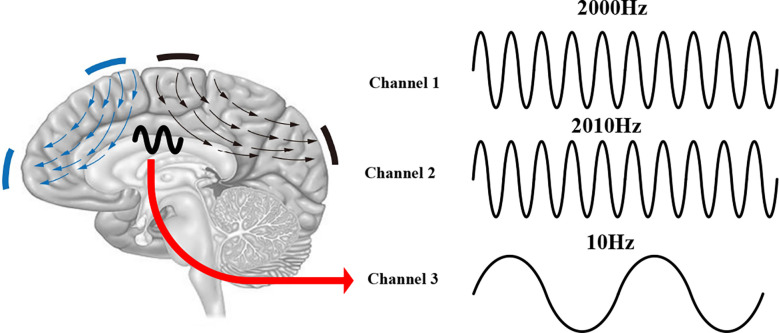
Schematic diagram of temporally interfering (TI) stimulation.

If the frequency of AM-tACS is further increased to the kHz level and the return electrodes of AM-tACS are separated, then the configuration is the embryonic form of TI stimulation (Figure 5). Grossman verified the TI stimulation technique through a series of experiments. By detecting the expression of c-fos protein in the brains of mice after TI stimulation, it was verified that TI stimulation could stimulate the deep part of the brain without affecting the cerebral cortex. Then, the ability to control and focus the spatial position of TI stimulation was verified in *in vivo* experiments (Grossman et al., 2017). The principle of TI stimulation lies in the high-frequency non-response characteristics of neurons verified by Grossman. What truly affects the neurons in the brain is not the two high-frequency electrical signals applied but a low-frequency electrical signal obtained by coupling two high-frequency electrical signals. This low-frequency electrical signal can affect the activity of the brain electrical signal corresponding to its frequency. Since the development of this technique, although there have been many studies on TI stimulation modeling, there have been few animal experiments. Moreover, whether the depth of TI stimulation can reach the centimeter level needs to be verified, and there is no research on the application of TI stimulation to the human body. More research is still needed at the mechanism level on aspects such as whether the high-frequency non-response phenomenon is correct (Opitz et al., 2017). At present, TI stimulation methods are mainly used in modeling and animal experiments; even animal experiments are rarely discussed, which shows that TI stimulation methods still have a long way to go in the transition from research to application.

### Intersectional Short Pulse Stimulation

**Figure 6:**
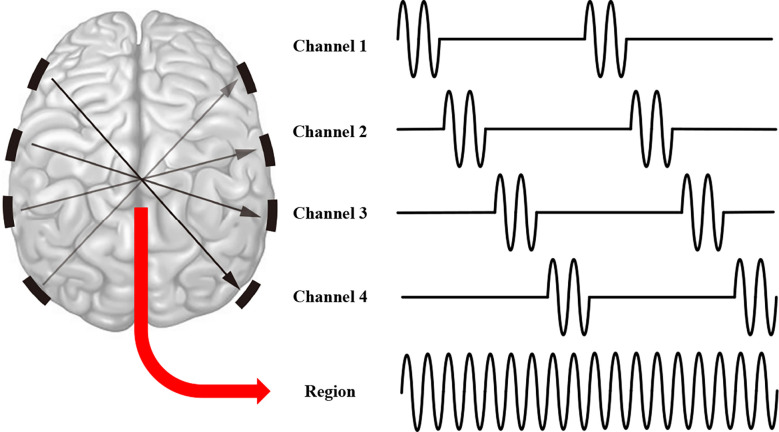
Schematic diagram of intersectional short pulse (ISP) stimulation.

From phase-shifted tACS, we found that different phases of tACS are effective. Regarding the TI stimulation mode, we found that the two groups of electrodes can stimulate the same position by setting the electrode position and stimulation parameters. What would happen if we did not add brain regions to be stimulated, but instead used stimulating signals of different phases at the same point at different times? Transcranial stimulation at different times means that each electrode position is not stimulated for a long time, so we can increase the intensity of stimulation on the electrode in each cycle without having too much of an effect on the cortex of the electrode location. Therefore, the brain can withstand higher-intensity stimulation. ISP stimulation is an improved method based on this idea. Its working principle is the same as that of ordinary tACS; that is, the frequency of electrical signal application affects the discharge activity of neurons in the same channel in the brain. The main improvement of ISP stimulation is that a complete stimulation cycle is divided into several parts, each of which is completed by a pair of electrodes. By increasing the stimulation electrodes, the discharge time of each stimulation electrode in the skin is reduced, thus reducing all kinds of adverse effects that may be caused by stimulation of the skin for a long time by current. The schematic diagram of ISP stimulation is shown in Figure 6. Multiple electrode pairs are used to alternately transmit stimulation current, and the surface layer of the brain is stimulated discontinuously in time, so even the use of high-amplitude current stimulation does not cause safety problems. On the other hand, if the stimulation positions of each electrode in the deep part of the brain overlap, the deep part of the brain receives continuous stimulation in time and undergoes high-intensity stimulation. Because ISP stimulation is still in the research stage, its application frequency and amplitude are still in the research stage, but its application stimulation amplitude is much larger than that of traditional tACS; in human experiments, the ISP stimulation amplitude can reach 7 mA (although dizziness occurs). Vöröslakos demonstrated the regional specificity of this new method in rodents; that is, neuron circuits are transiently affected by higher-intensity currents compared to those used in traditional schemes (Vöröslakos et al., 2018). However, 1-Hz ISP is not comfortable when applied to people and seems to lack a long-term post-stimulation effect (Widge, 2018).

## Computer Stimulation

Experiments are only part of the verification of the abovementioned optimization methods, and there are many studies that have explored the method and effect of optimization through computer simulation to explain the mechanism of optimization methods. Different models were established to determine whether electrical stimulation was effective and to optimize the number of electrodes and electrode arrangement (Morales-Quezada et al., 2019). Aberra established a computational model of human and rat cortical neurons to simulate the neural response of the cerebral cortex stimulated by electromagnetic fields (Aberra et al., 2018). Cakan and Obermayer (2020) used a simplified mean field model of adaptive exponential integrate-and-fire (AdEx) neurons to discuss the effects of electrical stimulation on large nerve populations (Cakan and Obermayer, 2020). At the same time, we should also note that computer simulation is useful but has many limitations. For example, they made assumptions about tissue conductivity, but different hypothetical values may lead to very different results in the size of the electric field (Laakso et al., 2015; Saturnino et al., 2015). Other factors that change the electric field include anatomical variations in functional connectivity and individual variability (Laakso et al., 2016). In addition, because different neurons have different excitability, the interaction between these neurons and extracellular stimulation is complex. Therefore, the biological mechanism of neuroregulatory methods such as tACS is not completely clear (Cubo et al., 2018), and many factors need to be analyzed to understand the effect of a given brain stimulation approach (Karimi et al., 2019). Therefore, this section summarizes the computer simulation results of electrode parameters, high-definition electrical stimulation, and interference modulation stimulation.

### Simulation of Electrode Parameters

The position and shape of the electrode have been proven to be directly related to the focus of electrical stimulation (Dmochowski et al., 2011; Bikson et al., 2013). Due to the uneven distribution of current or the shunt of current on the skin, it is very difficult to ensure the accurate placement of electrodes with sufficient distance between electrodes. This requires us to solve this problem through computer simulation technology.

Studies have shown that only focus montages such as circular montages or high-definition montages can specifically manipulate the phase relationship between two target regions without injecting unwanted electric fields into other regions (Saturnino et al., 2017; Karabanov et al., 2019). Moreover, the more electrode pairs there are, the more the activation region can be targeted accurately (Karimi et al., 2019). Compared with the classical electrode montage, the ring electrode montage can stimulate the target region more intensively and significantly reduce neurosensory side effects (Heise et al., 2016). In addition, studies found that circular electrodes have a better restraining effect on the electric field distribution of different phantom layers than rectangular electrodes (Morales-Quezada et al., 2019).

### High-Definition Electrical Stimulation

tACS can regulate the intrinsic neural activity of the brain by injecting a weak current into the electrodes connected to the scalp. High-resolution computational models show that cerebrospinal fluid conducts electricity to “hot spots”, depending on the specific anatomy of the skull and the specific folding of the brain; these features are not immediately visible from the location of the electrodes on the scalp (Datta et al., 2009, 2011). Pertinently, although the weak electric field with an intensity of 1 V/m usually used in tACS experiments has only a small effect on the membrane potential of a single neuron (Radman et al., 2009), the effect on the dynamics of the network may be quite significant; this effect has also been observed in an experiment (Francis et al., 2018). Therefore, it is necessary to model and optimize HD-tACS stimulation so that it can be applied more accurately (Dmochowski et al., 2011).

Ruffini first described a method of optimizing a multi-focus TES structure by the least square method and extended the application of this method from HD-tDCS to HD-tACS (Ruffini et al., 2014). Then, more people optimized HD-tACS. Cancelli used the finite element method to simulate the focus and intensity of different numbers of electrodes at the head and found that using only 2–8 electrodes can achieve reasonable target positioning, which provides theoretical support for the optimization of HD-tACS (Cancelli et al., 2016). During computer simulation, people found that during electrical stimulation, the electric field attenuation of the skin was several times larger than that of the skull, and they predicted that an electrical conductivity of 0.57 S/m would be the global value of the whole brain (Asan et al., 2019).

Subsequently, people have studied bilateral HD-tACS. Saturnino proposed an optimized HD-tACS electrode montage, which can better target the same region in both positive and negative phase conditions (Saturnino et al., 2017). Alekseichuk estimated the electric field of three different HD-tACS montages using real mouse, monkey, and human finite element models (Alekseichuk et al., 2019c). They found that the electric field intensity induced by HD-tACS is different in different samples, and the results they set up were consistent with those in the human experimental literature (Antal et al., 2017; Grossman et al., 2018).

### Interference Modulation Electrical Stimulation

The case of interference modulation is more complex because the principle of interference modulation is that two or more signals influence each other to produce the desired waveform. There are two areas that need modeling and verification. Whether two or more signals can correctly interfere with each other in the brain to become the signal we want is an important aspect of this method. It is unclear how the electrodes should be arranged so that the position of the interference modulation can be accurately controlled.

The main advantage of AM-tACS is that it can reduce stimulus artifacts. However, the basic mechanism of the involvement of AM-tACS waveforms in neuronal targets is still unclear. Ehsan used a computational model of the cerebral cortex to study how AM-tACS modulates endogenous oscillations and found that AM-tACS needs a much higher current intensity than the unmodulated tACS waveform to achieve obvious phase synchronization (Negahbani et al., 2018). In addition, AM-tACS theoretically does not need power at its modulation frequency, which avoids the problem of spectrum overlap between brain signals of interest and stimulus artifacts. However, through computer simulation, Kasten found that even the weak nonlinearity of the stimulation and recording hardware also lead to disordered modulation of the frequency and its harmonics, and artifacts are still present in the stimulation process (Kasten et al., 2018). Therefore, in view of the artifacts still existing in AM-tACS, David successfully separated the stimulus artifacts in AM-tACS through a real-time compatible artifact suppression algorithm and verified it in seven healthy volunteers (Haslacher et al., 2020).

As a new type of non-invasive deep brain stimulation, TI stimulation has attracted much attention. The study of TI stimulation is being promoted in different ways. Lee demonstrated the effectiveness of the TI method and its feasibility in detecting relatively large electrical conductivities in human tissues by numerical and experimental results (Lee et al., 2018). Cao simulated the membrane potential response of neurons to TI stimulation through the HH model (Cao and Grover, 2020). Furthermore, Cao and Xiao simulated multi-electrode TI stimulation to explain a series of principal problems regarding interference current and spatial resolution (Xiao et al., 2018; Cao and Grover, 2020). However, their research still has some shortcomings in the physiological model. The HH model was also used to determine whether TI stimulation is effective for all types of neurons or whether it is the network mechanism that makes TI stimulation possible (rather than individual neuron dynamics; Cao and Grover, 2018).

After the verification of TI stimulation, people began to optimize it from different aspects. Lee optimized the configuration of scalp electrodes and injected current during TI stimulation to provide the maximum deep brain stimulation current (Lee et al., 2020). Rampersad proposed an optimal four-electrode current mode to maximize the TI electric field in the globus pallidus (0.37 V/m), hippocampus (0.24 V/m), and motor cortex (0.57 V/m; Rampersad et al., 2019). On the basis of TI stimulation, Zhu introduced and verified the concept of multi-point time interference (MTI) stimulation, which can stimulate multiple nodes in the brain network at the same time to regulate the function of the network (Zhu et al., 2019). Focusing on the uniform model with two and four electrode pairs, Karimi proposed two automatic algorithms for estimating stimulus parameters using an artificial neural network (ANN; Karimi et al., 2019). Based on TI stimulation, Cao studied the mechanisms of two-electrode-pair TI stimulation and then proposed “Spatio-Temporal Interference-based Stimulation Focusing Strategies” (STIMULUS) to improve the spatial precision of stimulation (Cao and Grover, 2020). Song simulated the typical electrode configurations of opposite electrodes and cross electrodes. The results show that the envelope modulation amplitude distribution of different electrode configurations is different in the same electric field direction (Song et al., 2019).

There are few studies on ISP stimulation and phase-shifted tACS. The main result of phase-shifted tACS is shown in “Phase-Shifted tACS” section. In addition, studies have confirmed that the three optimized methods (HD-tACS, TI, and ISP) are largely equivalent in maximizing the intensity or focus of the desired target position (Huang and Parra, 2018).

## Hardware

Due to the particularity of the number of electrodes and stimulation signals of the above optimized electrical stimulation, in many cases, even if the modeling is smooth, there will be a variety of unpredictable situations due to hardware constraints in the actual experimental process. In this section, we summarize the recent innovations in electrical stimulation hardware and try to associate them with the above electrical stimulation (HD-tACS, phase-shifted tACS, AM-tACS, TI, and ISP) to provide some ideas for better applications of these electrical stimulations.

For HD-tACS, the stimulation effect is different with different capacitance structures. Therefore, Dos Santos proposed a coplanar capacitive electrode structure. This capacitance technology can generate a large number of different stimuli by changing waveforms, amplitudes, frequencies, and cycles (Dos Santos et al., 2019). The interference modulation method is very suitable for the standard low-voltage CMOS process. At the same time, the HD-tACS electrode can also be designed with new composite materials. Spyropoulos proposed a plant composite electrode material based on aloe hydrogels and conductive polymers. The authors found that the biocompatibility and stability of these electrodes were better than those of traditional electrodes by placing this electrode on rat skulls (Spyropoulos et al., 2019a).

High-frequency stimuli with frequencies ≥1 kHz have received increasing interest in neuroregulation because their physiological and clinical features suggest a mechanism of unconventional nerve stimulation (Noori and Neel, 2018; Thomson et al., 2018). Traditional stimulus voltage and current isolators do not support the generation of such waveforms because they introduce biases. Fallahrad illustrates a custom device design that supports reliable electrophysiological recording during kHz frequency stimulation (Fallahrad et al., 2019). At high frequencies (for example, 2 kHz), scalp impedance is at least one order of magnitude lower than that of conventional stimulation (5–200 Hz; Pazhouhandeh et al., 2019). This is also a research direction for TI stimulation. In addition, Spyropoulos introduced an internal ion-gated organic electrochemical transistor (IGT), which can be locally magnified directly at the device–scalp interface. This IGT can reduce the contact size by five orders of magnitude, and the equipment can be easily installed between hair follicles, which greatly simplifies placement. If we want to make electrical stimulators safe, suitable for long-term implantation, or wearable in the future, this kind of IGT will have good application (Spyropoulos et al., 2019b).

## Discussion and Future

### Existing Problems or Challenges

To date, the current research progress of these different electrical stimulation methods is also different. Based on the available literature, we summarized the characteristics of different improvements and their advantages and disadvantages, as shown in Table 3. There is no conclusive evidence that transcranial electrical stimulation can damage brain tissue or cognitive function. However, there are still many challenges that need to be overcome in the clinical application of these improved methods. Because tACS is basically a non-invasive form of brain stimulation, some researchers question whether this method can truly work in the larger human brain as tissue size increases. The applied current intensity may need to be very high. If this is true, people will probably have to undergo percutaneous puncture under anesthesia for proper stimulation, limiting the potential application to some extent (Caulfield and George, 2018).

**Table 3:** Comparison of different tACS improvement methods.

Method	Purpose of improvement	Number of stimulation electrodes	Stimulus signal parameters		Stimulus focus	Stimulus intensity	Stimulation artifact	Stimulating effect
			Frequency	Amplitude				
HD-tACS	Increase targeting accuracy	4 cathode and 1 anode or 4 anode and 1 cathode	Alpha band and theta band	1–2 mA	Precision	Medium	Medium	Compared with that of traditional tACS, the stimulation intensity of the stimulation area is higher and the stimulation is more focused
Phase-shifted tACS	Change the stimulus phase to affect the connectivity of the stimulated brain region	3	Alpha band and theta band	1–2 mA	General	Medium	Medium	The magnitude of the electric field is different under different stimulation phases, and the electric field intensity under the reversed-phase condition is significantly higher than that under the same phase stimulation condition (2–2.3 times).
AM-tACS	Reduce stimulus artifacts	3	Modulated signal: Alpha band and theta band. Carrier: hundreds of Hz	1–2 mA	General (existing diffuse stimulation)	Medium	Low	Stimulation artifacts can be reduced to the extent that tACS is not applied
Temporally interfering stimulation	Reduce stimulus diffusion and stimulate directly into deep brain areas.	4	Thousands of Hz	100–400 μA (mice)	Precision	Low	Low	Through interference, stimulation can be directly applied to the region of interest to reduce the effect on the cortex, and the focus of the stimulation can be changed by changing the stimulation current rather than the position of the electrode.
Intersectional Short Pulse Stimulation	Enhance focus and increase the amplitude of the stimulus	Multiple pairs of electrodes	1–1,000 Hz	1–7.5 mA	General (existing diffuse stimulation)	High	Medium	The current intensity can be much higher than that of the above stimulation methods while keeping the charge density and sensation on the scalp surface relatively low.

Doubts about HD-tACS are the same as those about tACS because high-definition electrical stimulation only enhances the focus of stimulation; otherwise, the principle is the same as that of tACS. The mechanism of this kind of electrical stimulation may involve various synaptic and non-synaptic effects on neurons, non-neuronal cells, and tissues of the central nervous system, and it requires further exploration (Brunoni et al., 2012).

For interfering modulation, there is little research on modeling in AM-tACS, and no articles had been published on human experiments until this year. This makes people doubt the effectiveness of this method. However, most related studies have reported that AM-tACS does not completely prevent the interference of artifacts as it was expected to do. Therefore, AM-tACS research and traditional tACS research may also be plagued by artifacts; this may also be a future research direction of AM-tACS. For phase-shifted tACS, the main problem is whether it can achieve the effect of computational simulation *in vivo*; this aspect remains to be studied. For TI stimulation, the main problems are as follows. One problem is that it is unclear how to change the electric field distribution when the input currents of the two electrode pairs are not equal. Another problem is that whether the quasi-static hypothesis of basic physics is applicable to stimuli in the range of kHz must be verified (Huang and Parra, 2018). ISP stimulation faces the same problem as TI stimulation. In addition, many of the above stimulation methods are only used in animal research but not in the clinic, mainly because the depth of these stimulation methods has not been verified. Normal tACS can be seen as a diffuse stimulus, and these improvements can be seen as non-invasive deep brain stimulation. Therefore, we do not know whether the interference of electrical stimulation can be extended to the human brain, which is approximately 1,400 times the size of a mouse brain. Whether the neural elements in the stimulation pathway are truly unaffected in the larger brain remains to be determined (Lozano, 2017).

### Dose and Safety

Researchers should also try to understand the best therapeutic “dosage” of electrical stimulation. The concept of electrical stimulation “dosage” was first proposed by Nitsche and Paulus (2000) and various dose options also make it difficult to determine the optimal choice. For high-definition electrical stimulation, cortical areas exposed to high-dose electrical stimulation may be more likely to be modulated. However, other less controllable factors, such as skin and skull resistance, may also affect the effective flow of electricity to neuronal tissue. In the study of human transcranial electrical stimulation, the field intensity of a 1-mA current is between 0.2 V/m and 0.5 mV/m when it reaches the target site (Opitz et al., 2016; Huang et al., 2017). This magnitude of field strength is considered to be the minimum required for the human body to produce measurable physiological effects in neurons. A recent *in vitro* study showed that the skull and soft tissue around the brain diverted approximately 60% of the injected current 75% away from the brain (Vöröslakos et al., 2018). According to this study, the current administered to the brain in tACS is too low to directly produce measurable physiological effects but may affect the brain through rhythmic resonance (de Berker et al., 2013). Therefore, whether to increase the dose of electrical stimulation in future research and studying the effect of the dose of electrical stimulation are also important directions of future research on the mechanism of transcranial electrical stimulation.

At present, there are no adverse reactions when HD-tACS is used in the human body. For interference modulation stimulation, the situation is more complicated (Zhao et al., 2017). Most interference modulation methods have only been verified in rodents, so safety in humans remains to be verified, but some subjects have felt pain when ISP stimulation was tested in humans. This requires us to establish an optimal current and electrodes to make electrical stimulation both physiologically effective and safe in the human body. In addition, for interference modulation, the spatial target of the interference field in the deep structure of the human brain will be further complicated by the anatomy of the head and brain tissue. The orientation method based on multiple stimulation electrodes originally developed for a low-frequency electric field needs to be extended and verified before it can be applied to a high-frequency field (Opitz and Tyler, 2017). In addition, it should be noted that a variety of factors may affect the ultimate clinical efficacy of the optimized tACS method in psychological and neurological diseases; further studies should focus on individual differences in specific diseases (for example, mental state, treatment resistance, and disease severity; Kekic et al., 2016). This requires us to pay special attention to the design of experimental groups when designing experiments to verify the optimized methods of tACS. In addition, recent studies reported that participants could distinguish between sham stimulation and transcranial current stimulation (Greinacher et al., 2019). Therefore, we should pay special attention to the design of the control group when designing a clinical trial.

### Ethics

Before the clinical application of these alternating current stimulation methods, we should maintain a good critical attitude towards these new technologies. Even non-invasive neuroregulatory techniques are not toys and can have unintended neuropsychological consequences (Bikson et al., 2013). We should support the standardized sociology of biological knowledge, which can benefit from the principles of justice, kindness, and non-harm, as well as the concept of human rights (Petersen, 2013). Since we currently do not understand the intricacies of brain regulation and its effects on cognitive processes and body function, we should at least verify that these methods are harmless before applying them. This requires us not only to conduct further preclinical research in relevant animal models but also to verify the outcomes with brain imaging methods to prevent possible adverse results. The main advantages of alternating current stimulation are that it is not invasive (non-invasive brain stimulation does not require general anesthesia, resulting in fewer complications than invasive brain stimulation), reversible (neuroregulation can be stopped immediately if there is a problem), and adaptable/flexible (brain targets and/or stimulation parameters can be easily and quickly modified; Val-Laillet et al., 2015). However, since many of the above methods are only based on experiments on rodents, we must accurately study them further to balance the costs and advantages of each method.

At present, one of the most commonly used directions of the above methods is to change memory ability through neural modulation. Surveys show that many college students use Ritalin and other “learning drugs” without a prescription, hoping to improve their attention and memory even if they are not diagnosed with mental disorders (Farah, 2015). If the above electrical stimulation is proven to have a modulating effect on memory function and is used clinically, it is likely to be used recklessly by people without proper guidance or information, resulting in potentially harmful effects. In addition, alternating current electrical stimulation as a form of non-invasive electrical stimulation may affect one’s mood, attention, reasoning, and social behavior (Hamilton et al., 2011). When the improved method is extended to clinical trials, we must consider the above aspects that occur after stimulation. Although the improved methods of alternating current electrical stimulation mentioned above are all designed to enhance the focus of stimulation, non-target stimulation is still a worrying problem (Davis and van Koningsbruggen, 2013). Changing cognition by targeting the structure of the brain can have unwanted side effects, reduce expected results, and even lead to contradictory reactions.

### Perspectives for the Future

Generally, there is no perfect explanation of the mechanism to prove whether the above improved tACS methods are effective. First, animal experiments are essential. Key mechanistic insights can be obtained from animal studies that examine different forms of electrical stimulation or investigate other results that cannot be achieved in humans (Zhao et al., 2017). Meanwhile, in the human body, comprehensive techniques such as transcranial magnetic resonance imaging (MRI), electroencephalogram (EEG) recordings, and functional magnetic resonance imaging (fMRI) can also help us to better understand the mechanism of electrical stimulation of the brain. Neuling points out that due to the electrical characteristics of tACS itself, the artifacts of tACS make it impossible to parse EEG signals under the condition of tACS (Neuling et al., 2016). Hyvarinen combined tACS with MEG and used MEG to observe auditory evoked steady-state activity under tACS conditions (Hyvarinen et al., 2018). In contrast, fMRI is the easiest method to combine with tACS. Neubauer combined θ tACS with fMRI to analyze the brain changes induced by prickles (Neubauer et al., 2017). Chen analyzed the role of 10-Hz and 20-Hz tACS in the integration and isolation of chronic stroke networks by fMRI (Chen et al., 2021). These studies point out a direction for us to explore the mechanism of tACS from various angles in the future. In addition, in human experiments, it is also important to choose different markers to explore the therapeutic effect of electrical stimulation on different diseases. Takeyama found that electrical stimulation could induce a unique positive evoked potential, called “widespread P1” (P1w). In the future, this newly evoked potential may provide a new opportunity to evaluate its clinical application as a biomarker of memory disorders in neurological diseases with various memory disorders, such as Alzheimer’s disease (Takeyama et al., 2019).

The current results show that to enhance the stimulation intensity and accuracy of tACS, the methods of improving tACS can be divided into high-definition electrical stimulation methods and interference modulation electrical stimulation methods. Since the mechanism of HD-tACS is consistent with that of tACS, there are many discussions about the application of HD-tACS in the human body. However, many interference modulation methods have not yet entered the stage of *in vivo* experiments or animal experiments. This is also a future research direction of the two methods. High-frequency tACS stimulation can be used to design retinal nerve stimulators, which have new applications in the treatment of visual loss (Barriga-Rivera et al., 2017). In addition, the optimized tACS method can be combined with other stimulation methods to achieve a better stimulation effect. For example, a new electrode was added to the HD-tACS method to change the electric field distribution in the stimulated cortex (Tashiro et al., 2020). Alternatively, visual stimulation can be combined with electrical stimulation to achieve higher spatial resolution and stimulate deep neurons (Xiao et al., 2019).

## Author Contributions

JW conceived the aim and contents of the review. LW meticulously analyzed the articles from the database queries to select the articles included in this review, and wrote the draft of the manuscript. TL developed the structure and integration of the contents of the review. All authors contributed to the article and approved the submitted version.

## Conflict of Interest

The authors declare that the research was conducted in the absence of any commercial or financial relationships that could be construed as a potential conflict of interest.

## References

[B1] Aberra A. S. Peterchev A. V. Grill W. M. (2018). Biophysically realistic neuron models for simulation of cortical stimulation. J. Neural Eng. 15:066023. 10.1088/1741-2552/aadbb1 30127100 PMC6239949

[B3] Alekseichuk I. Falchier A. Y. Linn G. Xu T. Opitz A. (2019a). Electric field dynamics in the brain during multi-electrode transcranial electric stimulation. Nat. Commun. 10:2573. 10.1038/s41467-019-10581-7 31189931 PMC6561925

[B4] Alekseichuk I. Mantell K. Shirinpour S. Opitz A. (2019b). Comparative modelling of transcranial magnetic and electric stimulation in mouse, monkey and human. NeuroImage 194, 136–148. 10.1016/j.neuroimage.2019.03.044 30910725 PMC6536349

[B6] Alekseichuk I. Turi Z. Veit S. Paulus W. (2019c). Model-driven neuromodulation of the right posterior region promotes encoding of long-term memories. Brain Stimul. 13, 474–484. 10.1016/j.brs.2019.12.019 31882373

[B5] Alekseichuk I. Pabel S. C. Antal A. Paulus W. (2017). Intrahemispheric theta rhythm desynchronization impairs working memory. Restor. Neurol. Neurosci. 35, 147–158. 10.3233/RNN-160714 28059806

[B8] Antal A. Alekseichuk I. Bikson M. Brockmöller J. Brunoni A. Chen R.. (2017). Low intensity transcranial electric stimulation: safety, ethical, legal regulatory and application guidelines. Clin. Neurophysiol. 128, 1774–1809. 10.1016/j.clinph.2017.06.001 28709880 PMC5985830

[B9] Asan A. S. Gok S. Sahin M. (2019). Electrical fields induced inside the rat brain with skin, skull and dural placements of the current injection electrode. PLos One 14:e0203727. 10.1371/journal.pone.0203727 30629578 PMC6328113

[B2] Barriga-Rivera A. Bareket L. Goding J. Aregueta-Robles U. A. Suaning G. J. (2017). Visual prosthesis: interfacing stimulating electrodes with retinal neurons to restore vision. Front. Neurosci. 11:620. 10.3389/fnins.2017.00620 29184478 PMC5694472

[B888] Bächinger M. Zerbi V. Moisa M. Polania R. Liu Q. Mantini D.. (2017). Concurrent tacs-fMRI reveals causal influence of power synchronized neural activity on resting state fMRI connectivity. J. Neurosci. 37, 4766–4777. 10.1523/JNEUROSCI.1756-16.2017 28385876 PMC6596494

[B7] Berger A. Pixa N. H. Steinberg F. Doppelmayr M. (2018). Brain oscillatory and hemodynamic activity in a bimanual coordination task following transcranial alternating current stimulation (tACS): a combined EEG-fNIRs study. Front. Behav. Neurosci. 12:67. 10.3389/fnbeh.2018.00067 29720935 PMC5915568

[B10] Bikson M. Bestmann S. Edwards D. (2013). Neuroscience: transcranial devices are not playthings. Nature 501:167. 10.1038/501167b 24025832 PMC4326099

[B11] Brunoni A. R. Nitsche M. A. Bolognini N. Bikson M. Wagner T. Merabet L.. (2012). Clinical research with transcranial direct current stimulation (tDCS): challenges and future directions. Brain Stimul. 5, 175–195. 10.1016/j.brs.2011.03.002 22037126 PMC3270156

[B12] Cakan C. Obermayer K. (2020). Biophysically grounded mean-field models of neural populations under electrical stimulation. PLos Comput. Biol. 16:e1007822. 10.1371/journal.pcbi.1007822 32324734 PMC7200022

[B13] Cancelli A. Cottone C. Tecchio F. Truong D. Q. Dmochowski J. Bikson M. (2016). A simple method for EEG guided transcranial electrical stimulation without models. J. Neural Eng. 13:036022. 10.1088/1741-2560/13/3/036022 27172063

[B14] Cao J. Grover P. (2020). STIMULUS: noninvasive dynamic patterns of neurostimulation using spatio-temporal interference. IEEE Trans. Biomed. Eng. 67, 726–737. 10.1109/TBME.2019.2919912 31150335

[B15] Cao J. Grover P. (2018). “Do single neuron models exhibit temporal interference stimulation?,” in 2018 IEEE Biomedical Circuits and Systems Conference (BioCAS). (Cleveland, OH: IEEE).

[B17] Caulfield K. A. George M. S. (2018). The future of brain stimulation treatments. Psychiatr. Clin. North Am. 41, 515–533. 10.1016/j.psc.2018.05.004 30098662

[B18] Chen C. Yuan K. Chu W. C.-W. Tong R. K.-Y. (2021). The effects of 10 Hz and 20 Hz tACS in network integration and segregation in chronic stroke: a graph theoretical fMRI study. Brain Sci. 11:377. 10.3390/brainsci11030377 33809786 PMC8002277

[B19] Cubo R. Åström M. Medvedev A. (2018). Optimization-based contact fault alleviation in deep brain stimulation leads. IEEE Trans. Neural Syst. Rehabil. Eng. 26, 69–76. 10.1109/TNSRE.2017.2769707 29324404

[B20] Datta A. Baker J. M. Bikson M. Fridriksson J. (2011). Individualized model predicts brain current flow during transcranial direct-current stimulation treatment in responsive stroke patient. Brain Stimul. 4, 169–174. 10.1016/j.brs.2010.11.001 21777878 PMC3142347

[B21] Datta A. Bansal V. Diaz J. Patel J. Reato D. Bikson M. (2009). Gyri-precise head model of transcranial direct current stimulation: improved spatial focality using a ring electrode versus conventional rectangular pad. Brain Stimul. 2, 201–207. 10.1016/j.brs.2009.03.005 20648973 PMC2790295

[B23] Davis N. J. van Koningsbruggen M. (2013). Non-invasive brain stimulation is not non-invasive. Front. Syst. Neurosci. 7:76. 10.3389/fnsys.2013.00076 24391554 PMC3870277

[B24] Dayan E. Censor N. Buch E. R. Sandrini M. Cohen L. G. (2013). Noninvasive brain stimulation: from physiology to network dynamics and back. Nat. Neurosci. 16, 838–844. 10.1038/nn.3422 23799477 PMC4876726

[B25] de Berker A. O. Bikson M. Bestmann S. (2013). Predicting the behavioural impact of transcranial direct current stimulation: issues and limitations. Front. Hum. Neurosci. 7:613. 10.3389/fnhum.2013.00613 24109445 PMC3790257

[B26] Deng Y. Reinhart R. M. Choi I. Shinn-Cunningham B. G. (2019). Causal links between parietal alpha activity and spatial auditory attention. eLife 8:e51184. 10.7554/eLife.51184 31782732 PMC6904218

[B27] Dmochowski J. P. Datta A. Bikson M. (2011). Optimized multi-electrode stimulation increases focality and intensity at target. J. Neural Eng. 8:046011. 10.1088/1741-2560/8/4/046011 21659696

[B28] Dos Santos M. P. S. Coutinho J. Marote A. Sousa B. Ramos A. Ferreira J. A. F.. (2019). Capacitive technologies for highly controlled and personalized electrical stimulation by implantable biomedical systems. Sci. Rep. 9:5001. 10.1038/s41598-019-41540-3 30899061 PMC6428833

[B29] Douglas Z. H. Maniscalco B. Hallett M. Wassermann E. M. He B. J. (2015). Modulating conscious movement intention by non-invasive brain stimulation and the underlying neural mechanisms. J. Neurosci. 35, 7239–7255. 10.1523/JNEUROSCI.4894-14.2015 25948272 PMC4420786

[B30] Fallahrad M. Zannou A. L. Khadka N. Prescott S. A. Stéphanie R. Zhang T. (2019). Electrophysiology equipment for reliable study of kHz electrical stimulation. J. Physiol. 597, 2131–2137. 10.1113/JP277654 30816558 PMC6462489

[B31] Farah M. J. (2015). The unknowns of cognitive enhancement. Science 350, 379–380. 10.1126/science.aad5893 26494744

[B32] Francis J. T. Gluckman B. J. Schiff S. J. (2018). Sensitivity of neurons to weak electric fields. J. Neurosci. 23, 7255–7261. 10.1523/JNEUROSCI.23-19-07255.2003 12917358 PMC6740448

[B34] Grossman N. Bono D. Dedic N. (2017). Noninvasive deep brain stimulation via temporally interfering electric fields. Cell 169, 1029–1041. 10.1016/j.cell.2017.05.024 28575667 PMC5520675

[B33] Greinacher R. Buhôt L. Möller L. Learmonth G. (2019). The time course of ineffective sham-blinding during low-intensity (1 mA) transcranial direct current stimulation. Eur. J. Neurosci. 50, 3380–3388. 10.1111/ejn.14497 31228880 PMC6899874

[B35] Grossman P. Alekseichuk I. de Lara G. Paneri K. Kunz P. Turi Z.. (2018). Transcranial direct current stimulation studies open database (tDCS-OD). bioRxiv [Preprint]. 10.1101/369215

[B36] Hamilton R. Messing S. Chatterjee A. (2011). Rethinking the thinking cap ethics of neural enhancement using noninvasive brain stimulation. Neurology 76, 187–193. 10.1212/WNL.0b013e318205d50d 21220723 PMC3030230

[B22] Haslacher D. Nasr K. Robinson S. E. Braun C. Soekadar S. R. (2020). Stimulation Artifact Source Separation (SASS) for assessing electric brain oscillations during transcranial alternating current stimulation (tACS). NeuroImage 228:117571. 10.1016/j.neuroimage.2020.117571 33412281 PMC7903161

[B37] Heise K. F. Kortzorg N. Saturnino G. B. Fujiyama H. Cuypers K. Thielscher A. (2016). Evaluation of a modified high-definition electrode montage for transcranial alternating current stimulation (tACS) of pre-central areas. Brain Stimul. 9, 700–704. 10.1016/j.brs.2016.04.009 27160465

[B38] Helfrich R. F. Knepper H. Nolte G. Struber D. Rach S. Herrmann C. S. (2014). Selective modulation of interhemispheric functional connectivity by HD-tACS shapes perception. PLos Biol. 12:e1002031. 10.1371/journal.pbio.1002031 25549264 PMC4280108

[B39] Huang Y. Parra L. C. (2018). Can transcranial electric stimulation with multiple electrodes reach deep targets? Brain Stimul. 12, 30–40. 10.1016/j.brs.2018.09.010 30297323 PMC6301116

[B40] Huang Y. Liu A. A. Lafon B. Friedman D. Dayan M. Wang X.. (2017). Measurements and models of electric fields in the *in vivo* human brain during transcranial electric stimulation. eLife 6:e18834. 10.7554/eLife.18834 28169833 PMC5370189

[B41] Hyvarinen P. Choi D. Demarchi G. Aarnisalo A. A. Weisz N. (2018). tACS-mediated modulation of the auditory steady-state response as seen with MEG. Hear. Res. 364, 90–95. 10.1016/j.heares.2018.03.023 29655974

[B42] Kadir S. Kaza C. Weissbart H. Reichenbach T. (2020). Modulation of speech-in-noise comprehension through transcranial current stimulation with the phase-shifted speech envelope. IEEE Trans. Neural Syst. Rehabil. Eng. 28, 23–31. 10.1109/TNSRE.2019.2939671 31751277 PMC7001147

[B43] Karabanov A. N. Saturnino G. B. Thielscher A. Siebner H. R. (2019). Can transcranial electrical stimulation localize brain function. Front. Psychol. 10:213. 10.3389/fpsyg.2019.00213 30837911 PMC6389710

[B44] Karimi F. Attarpour A. Amirfattahi R. Nezhad A. Z. (2019). Computational analysis of non-invasive deep brain stimulation based on interfering electric fields. Phys. Med. Biol. 64:235010. 10.1088/1361-6560/ab5229 31661678

[B45] Kasten F. H. Ehsan N. Flavio F. Herrmann C. S. (2018). Non-linear transfer characteristics of stimulation and recording hardware account for spurious low-frequency artifacts during amplitude modulated transcranial alternating current stimulation (AM-tACS). NeuroImage 179, 134–143. 10.1016/j.neuroimage.2018.05.068 29860086

[B46] Kekic M. Boysen E. Campbell I. C. Schmidt U. (2016). A systematic review of the clinical efficacy of transcranial direct current stimulation (tDCS) in psychiatric disorders. J. Psychiatr. Res. 74, 70–86. 10.1016/j.jpsychires.2015.12.018 26765514

[B47] Khatoun A. Breukers J. Beeck S. O. D. Nica I. G. Aerts J. M. Seynaeve L. (2018). Using high-amplitude and focused transcranial alternating current stimulation to entrain physiological tremor. Sci. Rep. 8:4927. 10.1038/s41598-018-23290-w 29563594 PMC5862845

[B48] Kuo H. I. Bikson M. Datta A. (2013). Comparing cortical plasticity induced by conventional and high-definition 4 × 1 ring tDCS: a neurophysiological study. Brain Stimul. 6, 644–648. 10.1016/j.brs.2012.09.010 23149292

[B49] Laakso I. Tanaka S. Koyama S. De Santis V. Hirata A. (2015). Inter-subject variability in electric fields of motor cortical tDCS. Brain Stimul. 8, 906–913. 10.1016/j.brs.2015.05.002 26026283

[B50] Laakso I. Tanaka S. Mikkonen M. Koyama S. Sadato N. Hirata A. (2016). Electric fields of motor and frontal tDCS in a standard brain space: a computer simulation study. NeuroImage 137, 140–151. 10.1016/j.neuroimage.2016.05.032 27188218

[B51] Lafon B. Henin S. Huang Y. Friedman D. Melloni L. Thesen T. (2017). Low frequency transcranial electrical stimulation does not entrain sleep rhythms measured by human intracranial recordings. Nat. Commun. 8:1199. 10.1038/s41467-017-01045-x 29084960 PMC5662600

[B52] Lang S. Gan L. S. Alrazi T. Monchi O. (2019). Theta band high definition transcranial alternating current stimulation, but not transcranial direct current stimulation, improves associative memory performance. Sci. Rep. 9:8562. 10.1038/s41598-019-44680-8 31189985 PMC6561937

[B54] Lee S. Lee C. Park J. Im C. H. (2020). Individually customized transcranial temporal interference stimulation for focused modulation of deep brain structures: a simulation study with different head models. Sci. Rep. 10:11730. 10.1038/s41598-020-68660-5 32678264 PMC7366675

[B53] Lee K. M. Li J. Bai K. (2018). “A novel current-interference scanning method for detection of abnormal tissues,” in ASME 2018 Dynamic Systems and Control Conference, (Atlanta, GA), V002T24A008.

[B55] Morales-Quezada L. El-Hagrassy M. M. Costa B. Mckinley R. A. Fregni F. (2019). Transcranial direct current stimulation optimization-from physics-based computer simulations to high-fidelity head phantom fabrication and measurements. Front. Hum. Neurosci. 13:388. 10.3389/fnhum.2019.00388 31736732 PMC6837166

[B56] Negahbani E. Kasten F. H. Herrmann C. S. Frohlich F. (2018). Targeting alpha-band oscillations in a cortical model with amplitude-modulated high-frequency transcranial electric stimulation. NeuroImage 173, 3–12. 10.1016/j.neuroimage.2018.02.005 29427848 PMC5911251

[B57] Neubauer A. C. Wammerl M. Benedek M. Jauk E. Jaušovec N. (2017). The influence of transcranial alternating current stimulation (tACS) on fluid intelligence: an fMRI study. Pers. Individ. Diff. 118, 50–55. 10.1016/j.paid.2017.04.016 29176918 PMC5700801

[B58] Neuling T. Ruhnau P. Weisz N. Herrmann C. S. Demarchi G. (2016). Faith and oscillations recovered: on analysing EEG/MEG signals during tACS. NeuroImage 147, 960–963. 10.1016/j.neuroimage.2016.11.022 27888060

[B59] Nguyen J. Deng Y. Reinhart R. M. G. (2018). Brain-state determines learning improvements after transcranial alternating-current stimulation to frontal cortex. Brain Stimul. 11, 723–726. 10.1016/j.brs.2018.02.008 29482970 PMC6019559

[B60] Nitsche M. Paulus W. (2000). Excitability changes induced in the human motor cortex by weak transcranial direct current stimulation. Physiology 527, 633–639. 10.1111/j.1469-7793.2000.t01-1-00633.x 10990547 PMC2270099

[B61] Noori S. Neel M. (2018). Management of medically refractory central poststroke pain using high-frequency spinal cord stimulation at 10 KHz. Neuromodulation 21, 823–825. 10.1111/ner.12718 29350884

[B62] Opitz A. Falchier A. Linn G. S. Milham M. P. Schroeder C. E. (2017). Limitations of ex vivo measurements for *in vivo* neuroscience. Proc. Natl. Acad. Sci. U S A 114, 5243–5246. 10.1073/pnas.1617024114 28461475 PMC5441777

[B63] Opitz A. Falchier A. Yan C. G. Yeagle E. M. Linn G. S. Megevand P. (2016). Spatiotemporal structure of intracranial electric fields induced by transcranial electric stimulation in humans and nonhuman primates. Sci. Rep. 6:31236. 10.1038/srep31236 27535462 PMC4989141

[B64] Opitz A. Tyler W. J. (2017). No implant needed. Nat. Biom. Eng. 1, 632–633. 10.1038/s41551-017-0120-y 31015602

[B65] Pazhouhandeh M. R. Orleary G. Weisspapir I. Groppe D. Genov R. (2019). “Adaptively clock-boosted auto-ranging responsive neurostimulator for emerging neuromodulation applications,” in 2019 IEEE International Solid-State Circuits Conference (ISSCC), (San Francisco, CA), 62, 374.

[B66] Petersen A. (2013). From bioethics to a sociology of bio-knowledge. Soc. Sci. Med. 98, 264–270. 10.1016/j.socscimed.2012.12.030 23434118

[B67] Lozano A. M. (2017). Waving hello to noninvasive deep-brain stimulation. N. Engl. J. Med. 377, 1096–1098. 10.1056/NEJMcibr1707165 28902594

[B68] Popp F. Dallmer-Zerbe I. Philipsen A. Herrmann C. S. (2019). Challenges of p300 modulation using transcranial alternating current stimulation (tACS). Front. Psychol. 10:476. 10.3389/fpsyg.2019.00476 30890990 PMC6411790

[B69] Radman T. Ramos R. L. Brumberg J. C. Bikson M. (2009). Role of cortical cell type and morphology in subthreshold and suprathreshold uniform electric field stimulation *in vitro*. Brain Stimul. 2, 215–228. 10.1016/j.brs.2009.03.007 20161507 PMC2797131

[B70] Rampersad S. Roig-Solvas B. Yarossi M. Kulkarni P. P. (2019). Prospects for transcranial temporal interference stimulation in humans: a computational study. NeuroImage 202:116124. 10.1016/j.neuroimage.2019.116124 31473351 PMC6819277

[B71] Reinhart R. M. G. (2017). Disruption and rescue of interareal theta phase coupling and adaptive behavior. Proc. Natl. Acad. Sci. U S A 114, 11542–11547. 10.1073/pnas.1710257114 29073084 PMC5664527

[B72] Ruffini G. Fox M. D. Ripolles O. Miranda P. C. Pascual-Leone A. (2014). Optimization of multifocal transcranial current stimulation for weighted cortical pattern targeting from realistic modelling of electric fields. NeuroImage 89, 216–225. 10.1016/j.neuroimage.2013.12.002 24345389 PMC3944133

[B74] Saturnino G. B. Antunes A. Thielscher A. (2015). On the importance of electrode parameters for shaping electric field patterns generated by tDCS. NeuroImage 120, 25–35. 10.1016/j.neuroimage.2015.06.067 26142274

[B75] Saturnino G. B. Madsen K. H. Siebner H. R. Thielscher A. (2017). How to target inter-regional phase synchronization with dual-site transcranial alternating current stimulation. NeuroImage 163, 68–80. 10.1016/j.neuroimage.2017.09.024 28919407

[B73] Saturnino G. B. Siebner H. R. Thielscher A. (2019). Accessibility of cortical regions to focal TES: dependence on spatial position, safety and practical constraints. NeuroImage 203:116183. 10.1016/j.neuroimage.2019.116183 31525498

[B76] Schwab B. C. Misselhorn J. Engel A. K. (2019). Modulation of large-scale cortical coupling by transcranial alternating current stimulation. Brain Stimul. 12, 1187–1196. 10.1016/j.brs.2019.04.013 31101568

[B77] Song X. Zhao X. Zhou Y. Liu S. Ming D. (2019). “Typical electrode configuration analysis for temporally interfering deep brain stimulation,” in 2019 9th International IEEE/EMBS Conference on Neural Engineering (NER), (San Francisco, CA), 770–773.

[B78] Spyropoulos G. D. Gelinas J. N. Khodagholy D. (2019a). Internal ion-gated organic electrochemical transistor: a building block for integrated bioelectronics. Sci. Adv. 5:eaau7378. 10.1126/sciadv.aau7378 30820453 PMC6392764

[B79] Spyropoulos G. D. Savarin J. Gomez E. F. Simon D. T. Khodagholy D. (2019b). Transcranial electrical stimulation and recording of brain activity using freestanding plant-based conducting polymer hydrogel composites. Adv. Mater. Technol. 5:1900652. 10.1002/admt.20190065230820453

[B80] Ströber D. Rach S. Trautmann-Lengsfeld S. A. Engel A. K. Herrmann C. S. (2014). Antiphasic 40 Hz oscillatory current stimulation affects bistable motion perception. Brain Topogr. 27, 158–171. 10.1007/s10548-013-0294-x 23709044

[B81] Takeyama H. Matsumoto R. Usami K. Nakae T. Ikeda A. (2019). Human entorhinal cortex electrical stimulation evoked short-latency potentials in the broad neocortical regions: evidence from cortico-cortical evoked potential recordings. Brain Behav. 9:e01366. 10.1002/brb3.1366 31361093 PMC6749511

[B82] Tashiro S. Siebner H. R. Charalampaki A. Gksu C. Tomasevic L. (2020). Probing EEG activity in the targeted cortex after focal transcranial electrical stimulation. Brain Stimul. 13, 815–818. 10.1016/j.brs.2020.02.015 32289712

[B83] Thomson S. J. Tavakkolizadeh M. Love-Jones S. Patel N. K. Gu J. W. Bains A.. (2018). Effects of rate on analgesia in kilohertz frequency spinal cord stimulation: results of the PROCO randomized controlled trial. Neuromodulation 21, 67–76. 10.1111/ner.12746 29220121 PMC5814855

[B84] Tomer F. Nikolaev A. R. Floris D. K. Aleksandra Z. Cees V. L. (2018). Multi-electrode alpha tACS during varying background tasks fails to modulate subsequent alpha power. Front. Neurosci. 12:428. 10.3389/fnins.2018.00428 29988498 PMC6026647

[B85] Tseng P. Iu K. C. Juan C. H. (2018). The critical role of phase difference in theta oscillation between bilateral parietal cortices for visuospatial working memory. Sci. Rep. 8:349. 10.1038/s41598-017-18449-w 29321584 PMC5762658

[B86] Val-Laillet D. Aarts E. Weber B. Ferrari M. Quaresima V. Stoeckel L. E.. (2015). Neuroimaging and neuromodulation approaches to study eating behaviour and prevent and treat eating disorders and obesity. Neuroimage Clin. 8, 1–31. 10.1016/j.nicl.2015.03.016 26110109 PMC4473270

[B87] Violante I. R. Li L. M. Carmichael D. W. (2017). Externally induced frontoparietal synchronization modulates network dynamics and enhances working memory performance. eLife 6:e22001. 10.7554/eLife.22001 28288700 PMC5349849

[B88] Vöröslakos M. Takeuchi Y. Brinyiczki K. (2018). Direct effects of transcranial electric stimulation on brain circuits in rats and humans. Nat. Commun. 9:483. 10.1038/s41467-018-02928-3 29396478 PMC5797140

[B89] Widge A. S. (2018). Cross-species neuromodulation from high-intensity transcranial electrical stimulation. Trends Cogn. Sci. 22, 372–374. 10.1016/j.tics.2018.03.006 29602532

[B90] Wischnewski M. Engelhardt M. Salehinejad M. A. Schutter D. J. L. G. Kuo M.-F. Nitsche M. A. (2018). NMDA receptor-mediated motor cortex plasticity after 20 Hz transcranial alternating current stimulation. Cereb. Cortex 29, 2924–2931. 10.1093/cercor/bhy160 29992259

[B91] Xiao Q. Lai X. Chen H. (2018). Multiple modulation synthesis with high spatial resolution for noninvasive deep neurostimulation. bioRXiv [Preprint]. 10.1101/416057 31220139 PMC6586313

[B92] Xiao Q. Zhong Z. Lai X. Qin H. (2019). A multiple modulation synthesis method with high spatial resolution for noninvasive neurostimulation. PLos One 14:e0218293. 10.1371/journal.pone.0218293 31220139 PMC6586313

[B93] Zhao H. C. Qiao L. Fan D. Q. Zhang S. Y. (2017). Modulation of brain activity with noninvasive transcranial direct current stimulation (tDCS): clinical applications and safety concerns. Front. Psychol. 8:685. 10.3389/fpsyg.2017.00685 28539894 PMC5423956

[B94] Zhu X. Q. Li Y. J. Zheng L. Shao B. X. Liu X. Li C. X. (2019). Multi-point temporal interference stimulation by using each electrode to carry different frequency currents. IEEE Access 7, 168839–168848. 10.1109/access.2019.2947857

[B95] Zoefel B. Allard I. Anil M. Davis M. H. (2019). Perception of rhythmic speech is modulated by focal bilateral transcranial alternating current stimulation. J. Cogn. Neurosci. 32, 226–240. 10.1162/jocn_a_01490 31659922 PMC7212037

